# The Role of Synovial Membrane in the Development of a Potential In Vitro Model of Osteoarthritis

**DOI:** 10.3390/ijms23052475

**Published:** 2022-02-24

**Authors:** Denisa Harvanova, Jana Matejova, Lucia Slovinska, Marek Lacko, Slavomira Gulova, Livia Kolesar Fecskeova, Jana Janockova, Timea Spakova, Jan Rosocha

**Affiliations:** 1 Associated Tissue Bank, Faculty of Medicine, Pavol Jozef Safarik University and Luis Pasteur University Hospital, Trieda SNP 1, 04011 Kosice, Slovakia; jana.matejova@upjs.sk (J.M.); lucia.slovinska@upjs.sk (L.S.); slavomira.gulova@student.upjs.sk (S.G.); livia.kolesar.fecskeova@upjs.sk (L.K.F.); jana.janockova@upjs.sk (J.J.); timea.spakova@upjs.sk (T.S.); jan.rosocha@upjs.sk (J.R.); 2 Department of Orthopedics and Traumatology of Locomotors Apparatus, Faculty of Medicine, Pavol Jozef Safarik University and Luis Pasteur University Hospital, Trieda SNP 1, 04011 Kosice, Slovakia; marek.lacko@upjs.sk

**Keywords:** cartilage, infrapatellar fat pad, in vitro model, osteoarthritis, synovial membrane

## Abstract

There is a lack of in vitro models able to plausibly represent the inflammation microenvironment of knee osteoarthritis (OA). We analyzed the molecules released from OA tissues (synovial membrane, cartilage, infrapatellar fat pad) and investigated whether the stimulation of human synovial fibroblasts (SFs), with synthetic cytokines (IL-1β and TNF-α or IFN-γ) or conditioned media (CM) from OA tissues, influence the SFs’ response, in the sense of pro-inflammatory cytokines, chemokines, growth factors, and degradative enzymes modulation. Human SFs were obtained from OA synovial membranes. SFs and their CM were analyzed for biomarkers, proliferation rate, protein profile and gene expression, before and after stimulation. Real-time PCR and multiplex assays quantified OA-related gene expression and biomolecule production. Unlike other activators, CM from OA synovial membrane (CM-SM), significantly up-regulated all genes of interest (IL-6, IL-8, MMP-1, MMP-3, RANTES, MCP-1, TSG-6, YKL-40) in SFs. Multiplex immunoassay analysis showed that levels of OA-related cytokines (IL-6, IL-8, MCP 1, IL-1Ra), chemokine (RANTES) and growth factor (VEGF), produced by CM-SM stimulated SFs, increased significantly compared to non-stimulated SFs. Molecules released from the SM from OA patients induces OA-like changes in vitro, in specific OA synovial populations (SFs). These findings promote the use and establish a compelling in vitro model that simulates the versatility and complexity of the OA disease. This model, in the future, will allow us to study new cell therapies or test drugs by reducing or avoiding animal models.

## 1. Introduction

In recent years, the definition of osteoarthritis (OA) has changed to whole joint disease. Knee OA affects the entire structure of the joints, including articular cartilage (CRT), synovial membrane (SM), subchondral bone, meniscus and infrapatellar fat pad (IFP) [1]. Typical structural characteristics of OA include CRT degradation, remodeling of subchondral bone, osteophyte formation, and changes in the synovium and joint capsule [2]. In the early stages of OA, proteoglycan and collagen degradation products are released from the hyaline CRT into the joint cavity. Structural changes in the joint induce catabolic mechanisms by chondrocytes and stimulate immune cells from the SM to release pro-inflammatory cytokines, mainly interleukin-1 beta (IL-1β), IL-6 and tumor necrosis factor α (TNF-α), and produce matrix metalloproteinases (MMP-1, -3, -13) and aggrecanases (ADAMTS-1, -2) [3]. MMP-13 and ADAMTS-5 are among the major catabolic enzymes involved in the breakdown of collagen and glycosaminoglycans (GAGs) in articular cartilage [4].

Synovial inflammation has been shown to act as a trigger for OA, leading to an increase in SM thickness and cellular infiltration of inflammatory cells, consisting of macrophages, lymphocytes, and natural killer (NK) cells [5,6]. Macrophages are mainly located in the synovial lining layer and their number notably increases, from low-grade to high-grade synovial inflammation. As mentioned earlier, the synovium excludes a number of catabolic and pro-inflammatory regulators during inflammation, such as TNF-α, IL-1β, IL-6, IL-8, IL-15, IL-17, IL-21, inflammatory mediators, such as prostaglandin E2 (PGE2), NO, adipokines (visfatin, resistin) and MMP-1, MMP-3, MMP-9, MMP-13, which promote cartilage degeneration and contribute to the progression of OA [7].

The synovium and IFP are considered an anatomical entity, actively involved in modulating intracapsular knee homeostasis. It is a site of immune cell infiltration and also as an active source of several pro-inflammatory/profibrotic and catabolic articular CRT mediators, including TNF-α, interferon-gamma (IFN-γ), connective tissue growth factor (CTGF) and MMPs [8,9].

Despite a number of studies, the understanding of the biological mechanisms involved in the pathogenesis of OA are still not clear [10]. To design new and improved treatments for OA, it is essential to understand the mechanism of disease diagnosis, progression, cellular and molecular factors, and the basic complex interaction in relevant tissues. Researchers are trying to find appropriate and valid preclinical models that simulate the versatility and complexity of the disease [11].

Up to date, many ex vivo, in vitro and in vivo models have been developed to mimic OA conditions. In vitro models provide more reproducible data, due to the ability to precisely control the experimental parameters and are less expensive and time consuming. Depending on the platform for cell culture and its interaction with the extracellular matrix (ECM), in vitro models can be divided into two-dimensional (2D) culture (monolayer or co-culture), 3D culture (scaffold-free or scaffold-based), explant-based culture and dynamic culture [12]. Novakofski et al. used a monolayer culture of chondrocytes, obtained from horse CRT, to study the effects of cytokine IL-1β, IL-6, or IL-8 stimulation [13]. Beekhuizen et al. developed and characterized an in vitro long-term CRT-synovium co-culture model. Both OA synovial tissue and CRT of the same knee from an OA patient were used for each co-culture to prevent direct cell–cell contact [14]. Jiang et al. designed a 3D co-culture model to study the interaction between chondrocytes and osteoblasts and to evaluate the influence of co-culture on the growing and phenotyping maintenance of mentioned cells [15]. Sanchez et al. generated a co-culture model, in which subchondral osteoblasts in the monolayer were cultured together with OA chondrocytes in alginate beads, to study the effect of OA osteoblasts on bone sclerosis and CRT degradation [16].

In general, 2D culture models allow for testing the effect of treatment at the cellular level and are a relatively cost-effective method to obtain rapid and reliable results. The 2D model involves the expansion of cells from a single source for multiple experimental treatments. Cell sources can vary from murine, bovine to human. Actually, the use of 2D culture models for OA is more appropriate for synoviocytes than chondrocytes. Chondrocytes tend to differentiate after a small number of passages. They change their phenotype and morphology from an orthogonal shape to an elongated shape that resembles fibroblast-like chondrocytes, leading to a reduction in the number of experiments [17].

Various biological and synthetic inflammatory stimuli can be used to induce OA-like conditions in vitro, which include, for example, (i) cytokines; (ii) synovial fluid from OA patients; (iii) conditioned media (CM) from the joint tissues of OA patients; (iv) CM from activated macrophages and others. Cytokines are the most commonly used components to induce inflammation, as they are inexpensive and can be easily adjusted and manipulated [11]. Cytokines play a key role in the progression of OA pathogenesis. They are responsible for the loss of metabolic homeostasis of the joint tissues by supporting catabolic and destructive processes.

IL-1β and TNF-α are among the two most commonly studied pro-inflammatory cytokines in OA [18]. IL-1β inhibits the synthesis of collagen type II and proteoglycans [19,20] and the level of IL-1β increases, in both early and late stages of OA. On the other hand, TNF-α acts at the beginning of the disease by triggering signaling pathways involved in tissue destruction. IL-1β and TNF-α regulate the inflammatory cascade independently or in conjunction with other cytokines [21]. IFN-γ, a major immune-modulating molecule, is expressed by bacterial and viral antigen-activated T-lymphocytes and NK cells. Conflicting evidence has been found regarding the pro-inflammatory and anti-inflammatory roles of IFN- γ [22,23], as deficiency of the IFN-γ or IFN-γ receptor (IFN-γR1) reduces or enhances susceptibility to collagen-induced arthritis. IFN-γ modulation and weakened endogenous IFN-γ response may be important mechanisms in OA pathogenesis and CRT degradation [24].

Explant-based models are simple, easy to fabricate and have the great advantage that they can be used to study the response of cells in their natural ECM. Eymard et al. showed that the CM of OA IFP can induce the production of IL-6 and IL-8, in addition to MMP-1, -3, and -9, in fibroblast-like synoviocytes isolated from the same patients with end-stage OA [25]. Belluzi et al. also demonstrated that CM from all OA joint tissues may be involved in the progression of synovitis, with the SM itself playing a predominant role compared to the other joint tissues [26].

**Figure 1:**
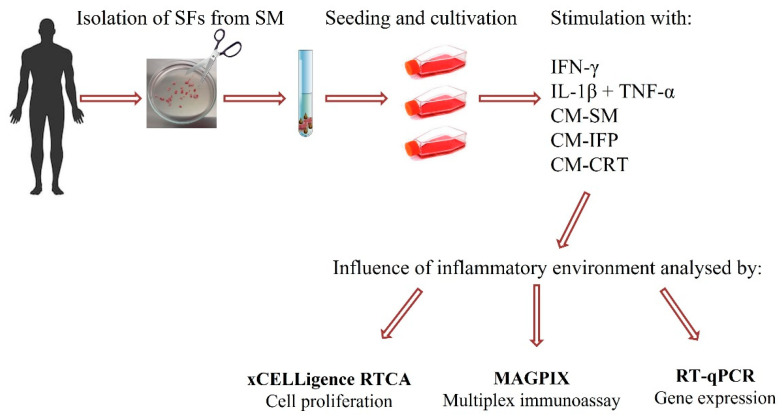
Schematic summary of manipulation, stimulation and analysis of SFs.

The aim of the present study was to establish a 2D in vitro model of OA. For this purpose, CM from OA joint tissues, such as SM, IFP and CRT, were prepared, analyzed, and subsequently used to stimulate synovial fibroblasts (SFs), isolated from OA SM. The effect of CM-SM, CM-IFP and CM-CRT on SFs activation was compared with the effect of synthetic cytokines (IL-1β + TNFα and IFN-γ). The impact of CM from OA joint tissues and synthetic cytokines on proliferation rate, release of pro-inflammatory molecules and gene expression by SFs was evaluated. The workflow of our experiments is shown in Figure 1.

## 2. Results

### 2.1. Phenotypic Characterization of SFs

Surface markers of SFs isolated from SM after passage 2 were analyzed by flow cytometry. The average results of the mean expression are presented in Table 1. Hematopoietic markers CD34, CD45 and CD14 were absent in cells (≤2%). Low expression was detected for CD106 (4.15%) and CD54 (33.58%). High expression was detected for CD26 (82.05%). CD105, CD73, CD90 were also highly expressed (≥90%).

### 2.2. Impact of Stimulation on SFs Proliferation Rate

**Figure 2:**
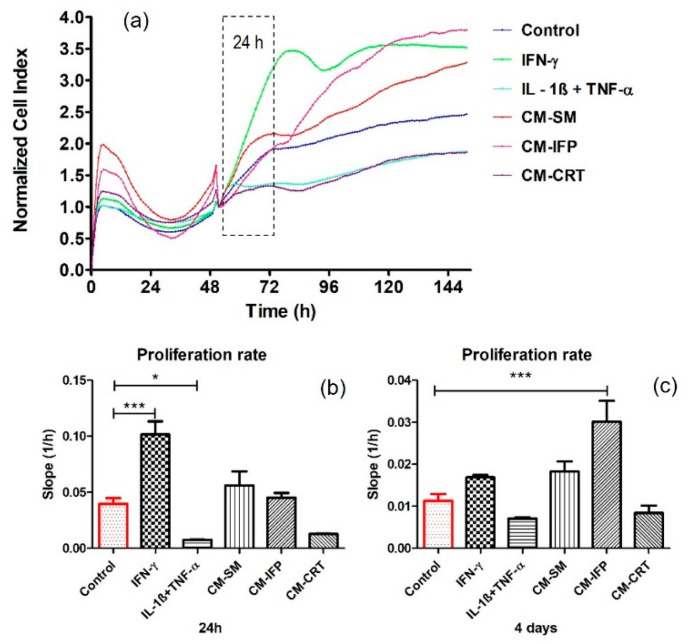
Real-time proliferation of SFs stimulated with CM from OA tissues and chemical cytokines: (**a**) proliferation curves (**b**) proliferation rate of SFs stimulated for 24 h; (**c**) proliferation rate of SFs stimulated 4 days. Data are presented as the mean ± SD (*n* = 5). Statistical significance (* *p* < 0.05, *** *p* < 0.001) for the particular experimental group compared to the control group (unstimulated SFs).

The effect of secreted factors released from OA joint tissues into CM and the impact of chemical cytokines (IL-1β + TNF-α, IFN-γ) on SFs proliferation were determined using xCELLigence^®^ system, which provides non-invasive electrical impedance monitoring to quantify cell proliferation, changes in morphology, and attachment quality in a label-free, real-time manner. In this work the proliferation rate of SFs after 24 h and 4 days of stimulation with OA joint tissues and cytokines (IL-1β + TNF-α, IFN-γ) was evaluated. Representative proliferation curves and proliferation rates are shown in Figure 2. IFN-γ significantly increased (*p* < 0.001) and the combination of IL-1β + TNF-α significantly decreased (*p* < 0.05) the proliferation of SFs compared to the control group, within 24 h stimulation. CM from OA joint tissues has no statistically significant impact on the proliferation of SFs (Figure 2b). Interestingly, a significant increase was observed in the proliferation rate (*p* < 0.001) of SFs stimulated with CM-IFP after 4 days. No changes in proliferation rate were observed in the other groups compared to the control Figure 2c.

### 2.3. Cytokine and Chemokine Release in CM from OA Tissues

**Figure 3:**
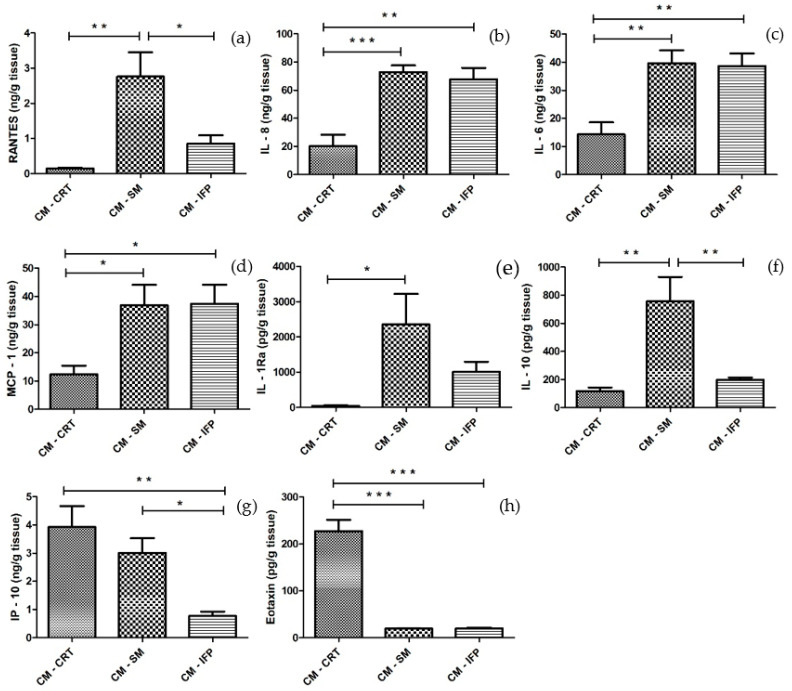
The concentrations of (**a**) RANTES; (**b**) IL-8; (**c**) IL-6; (**d**) MCP-1; (**e**) IL-1Ra; (**f**) IL-10; (**g**) IP-10 and (**h**) Eotaxin in CM-CRT, CM-SM and CM-IFP. Values are presented as the mean ± SD, (*n* = 5). Statistical significance (* *p* < 0.05, ** *p* < 0.01, *** *p* < 0.001) between the particular experimental groups.

The concentrations of cytokines and chemokines released into CM from OA tissues are shown in Figure 3. In CM-CRT, significant low levels of all molecules (RANTES, IL-8, IL-6, MCP-1, IL-1Ra, IL-10, Eotaxin), except IP-10, were detected compared to CM-SM. Significantly lower levels of IL-6, IL-8, MCP-1, IP-10, and Eotaxin, in CM-CRT, compared to CM-IFP, were also detected. Similar levels of IL-10 in CM-IFP and CM-CRT were found. On the other hand, CRT and SM produced significantly higher levels of IP-10 into CM (Figure 3g) compared to CM-IFP. The secretion of Eotaxin was significantly higher in CM-CRT in comparison to CM-SM and CM-IFP. Levels of growth factors (EGF, FGF, PDGF-AB/BB, VEGF) were low or no detectable in the CM of all joint tissues. 

### 2.4. Effect of CM from OA Joint Tissues and Cytokines on the Release of Pro-Inflammatory and Anti-Inflammatory Molecules by SFs

**Figure 4:**
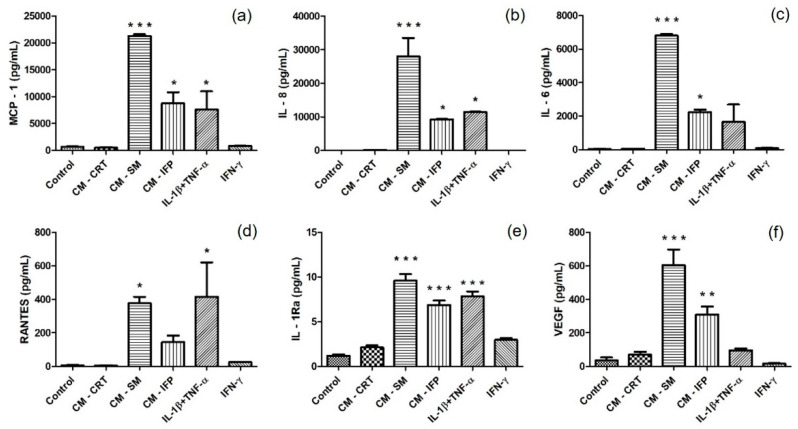
Secretion of (**a**) MCP-1; (**b**) IL-8; (**c**) IL-6; (**d**) RANTES; (**e**) IL-1Ra; and (**f**) VEGF in CM of unstimulated SFs (control) and in CM of SFs stimulated with CM-CRT, CM-SM, CM-IFP and cytokines (IL-1β + TNF-α, IFN-γ). The concentrations (pg/mL) are expressed as mean ± SD (*n* = 3). Statistical significance (* *p* < 0.05, ** *p* < 0.01, *** *p* < 0.001) for the particular experimental group compared to the control group (unstimulated SFs).

It was examined whether secretory factors from OA joint tissues can cause an inflammatory effect on SFs, in comparison with chemical cytokines. Protein release in CM after 24 h incubation of SFs with inflammatory agents was analyzed. Changes in the production of MCP-1, IL-6, IL-8, RANTES, IL-1Ra and VEGF in the CM of SFs were compared to levels in the CM from untreated SFs (control group) and are shown in Figure 4. In groups in which SFs were stimulated with CM-SM and CM-IFP, a significant increase in the concentration of cytokines IL-6, IL-8, MCP 1, IL-1Ra and growth factor VEGF were found. The activation of SFs with IL-1β + TNF-α caused the most significant changes in the production of IL-8, MCP 1, IL-1Ra and RANTES. Production of RANTES was significantly increased in group after stimulation with CM-SM (Figure 4d). CM-CRT and IFN-γ did not cause any changes in the production of inflammatory molecules compared to the control.

### 2.5. Gene Expression by Stimulated SFs

**Figure 5:**
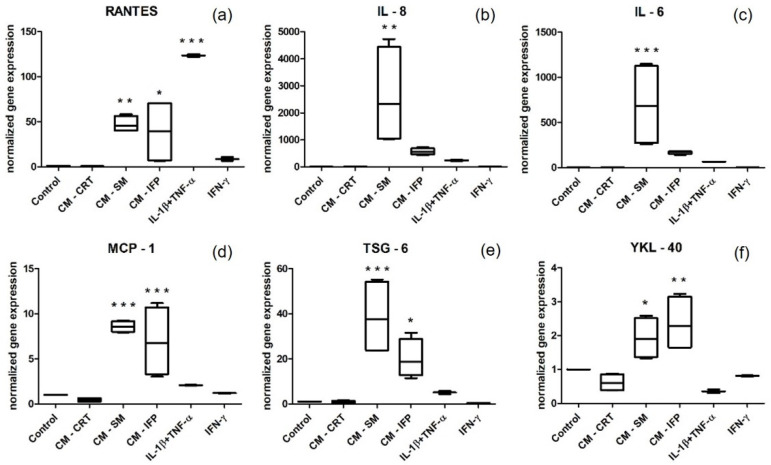

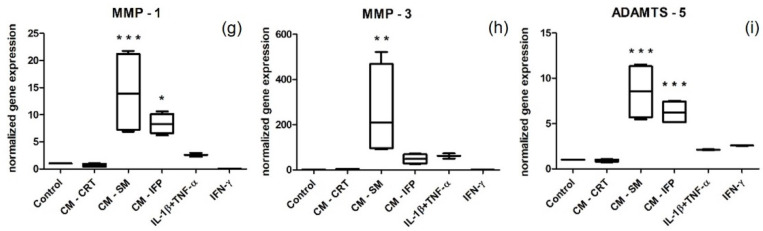
Normalized gene expression of pro-inflammatory markers (**a**) RANTES; (**b**) IL 8; (**c**) IL 6; (**d**) MCP-1; (**e**) TSG-6; (**f**) YKL-40; (**g**) MMP-1; (**h**) MMP-3; (**i**) ADAMTS-5 in SFs stimulated with CM of OA joint tissues (SM, IFP, CRT) and cytokines (IL-1β + TNF-α, IFN-γ). Levels of mRNA were normalized to two housekeeping genes (RPL13 and GAPDH). Results are the fold increase in gene expression relative to the unstimulated control (set at 1). Statistical significance (* *p* < 0.05, ** *p* < 0.01, *** *p* < 0.001) for the particular experimental group compared to the control group.

Stimulation of SFs with CM-SM and CM-IFP resulted in a statistically significant increase in the expression of all (in the case of CM-SM) (Figure 5) or almost all (in the case of CM-IFP) pro-inflammatory markers (except IL-8, IL-6, MMP-3) (Figure 5b,c,h) compared to the control. The expression of tested markers was significantly higher in the SFs stimulated with CM-SM than that of CM-IFP. However, stimulation with CM-CRT did not induce increased pro-inflammatory gene expression and mRNA levels were comparable to the unstimulated control. On the other hand, stimulation with chemical activators (IL-1β + TNF-α and IFN-γ) resulted in a statistically significant increase only in the expression of RANTES, compared to the control (Figure 5a).

## 3. Discussion

OA is a chronic disease of the entire joint, including structural changes in articular CRT, subchondral bone, SM and muscles around the joint. This degeneration process is characterized by the formation and development of a catabolic and inflammatory environment in the CRT and bone [27]. The causes of OA are still under consideration and up to now, no effective causal treatment is available to modify degenerative OA processes or prevent the progression of this disease. Since OA is a multifactorial disorder and its pathogenesis is difficult to understand, there is no complex model which could naturally demonstrate human OA. However, different ex vivo, in vitro, or in vivo models have been frequently used by researchers for the study of OA development and progression, but with no consensus on the most appropriate model. One of the most relevant is an in vitro inflammatory model, which plays an important role in elucidating biological processes in OA CRT and synovium, and allows for the testing of various therapeutic approaches [28]. When selecting an inflammatory OA model, many important aspects must be considered. The choice of a culture model (cell lines or primary cells, isolation methods, 2D/3D or cocultures) is a key parameter with great influence on tissue response [17]. 

In this work, a 2D in vitro model of OA was developed, with SFs stimulated by either recombinant cytokines (IL-1β + TNF-α, IFN-γ) or CM prepared from OA tissues, including SM, IFP, CRT. The effects of these various stimulations on cell proliferation, pro-inflammatory gene expression and pro-inflammatory and anti-inflammatory protein secretion were compared. It was found that stimulation of SFs, with CM of OA tissues, especially CM of SM, can induce a strong inflammatory environment, similar or even better than the stimulation by recombinant activators, and it could serve as a good in vitro model of OA.

In our study, SFs as the predominant cellular components of the joint synovium were used and their phenotype after passage 2 was analyzed by flow cytometry. There are not many specific and typical surface markers for fibroblasts and it is difficult to distinguish them from mesenchymal stem cells (MSCs). It was found, that SFs were negative (≤2%) for hematopoietic markers and highly expressed (≥90%) markers, typical for MSCs (CD105, CD73, CD90) [29]. CD26, as a fibroblast-specific surface marker, was also highly expressed (82.05%). CD106, which is generally absent in fibroblasts, had low expression (4.15%), as it was similarly described by Cappellesso-Fleury et al. [30]. Manferdini et al. showed that the isolation procedure of cells from the SM has an impact on the phenotypical and functional properties of SFs/macrophages. Synovial cells cultured in passage 1 contained both macrophages and fibroblasts, whereas only fibroblasts were preserved in passage 5 [31]. In our work, SFs showed low expression (1.45%) of the CD14 for synovial macrophages after passage 2 and fibroblasts formed most of the cell population. 

Tissue explants have the advantage of native ECM and spatial organization, and their use for evaluation of the cell responses is a simple and convenient method because the cells are located in a natural environment [11]. The availability of healthy clinical specimens is extremely limited. Clinical specimens are usually obtained from patients with advanced OA, but they may be contaminated with adipose tissue and are difficult to handle due to their irregular and deformed shape [28]. In our study, joint tissues, SM, IFP and CRT, from five patients with late-stage OA, were used and the molecules released into the CM were examined by using immunoassay kits. It was found that SM and IFP released comparably high amounts of the pro-inflammatory molecules IL-6, IL-8 and MCP-1 (Figure 5a,b,d). Deligne et al. demonstrated in their study, conducted among patients with OA, that IL-1β, IL-6, IL-8, IL-18, IL-17, IL-22 and TGF-β1 were increased in the inflammatory synovium tissues compared to the non-inflammatory tissues [32]. In addition, Favero et al. identified inflammatory molecules produced by co-cultivation of the meniscus and SM tissue from patients with early and end-stage OA. They demonstrated the presence of IL-6 and IL-8 in patients at both stages, but their levels were higher in the end-stage of OA [33]. Eymard et al. also found that IFP secreted higher levels of IL-6 and IL-8 compared to subcutaneous adipose tissue from the same OA patient [25]. On the other hand, in our study, SM produced significantly higher amounts of the immunomodulatory molecule RANTES and the anti-inflammatory molecules IL-1Ra and IL-10 compared to CRT (Figure 5a,e,f). In the study by Furuzawa-Carballeda et al., it was demonstrated that the synovium contains higher amounts of IL-6, IL-10 and the chemokines IL-8 and MCP-1 [34]. In our case, the levels of tested proteins released from the CRT into CM were low, except the chemokine Eotaxin and IP-10, which had the highest concentrations when compared to CM-SM and CM-IFP. The levels of chemokine IP-10 correlate with the severity of OA by playing a role in the settlement of leukocytes in inflamed tissues, which may then lead to the maintenance of chronic inflammation and resultant tissue damage. IP-10 has also been shown to play a role in modulating the response of chondrocytes to inflammatory stimuli associated with joint injury and the progression of post-traumatic arthritis [35].

Based on these results, high concentrations of pro-inflammatory cytokines were determined in the CM from all tested OA joint tissues. We hypothesized that these cytokines may stimulate SFs and induce an inflammatory milieu in vitro, in comparison to the chemical cytokines IL-1β, TNF-α and IFN-γ. Exogenous cytokines in in vitro systems belong to one of the most commonly used methods to induce inflammation. It is well known that inflammatory cytokines, such as IL-1β, TNF-α and IFN-γ, can be applied for modulation and imitation of the inflammatory catabolic environment of OA in vitro. TNF-α with IL-1β are considered as key inflammatory cytokines involved in the pathophysiological processes occurring in OA.

Multiplex Luminex bead-based analysis of the cell culture supernatant, from in vitro stimulated SFs, revealed different production of several cytokines, chemokines, and growth factors. It was demonstrated that the combination of chemical cytokines IL-1β + TNFα activated SFs and significantly increased the production of pro-inflammatory molecules MCP-1, IL6, IL8 and RANTES and IL-1Ra, compared to the control (Figure 4a–e), which is consistent with previous studies [18,36,37,38]. Stimulation of SFs with CM-IFP induced the production of comparable levels of cytokines as with chemical activators IL-1β + TNF-α (Figure 4a–e). In the study by Koroupis et al., IFP-MSC spheroids counter balanced the synoviocyte-induced inflammatory response via secretion of immunomodulatory molecules IP-10, MCP-1, MCP-2, RANTES, low IL-6/IL-8 ratio, as well as the articular CRT degradation inhibitor TIMP-2 [39]. In contrast, the cytokine IFN-γ and CM-CRT did not affect the production of tested inflammatory molecules by SFs. In the study by Utomo et al., upregulation of pro-inflammatory molecules IL-1β, IL-6, MMP-13, and ADAMTS-5 and downregulation of CRT matrix components, aggrecan and collagen type II, were observed in human osteoarthritic CRT explants cultured with the CM of pro-inflammatory macrophages, expressing IFN-γ and TNF-α [40]. The most significant changes in the production of all analyzed molecules, namely IL-6, IL-8, MCP-1, RANTES, IL-1Ra and VEGF, were observed after the stimulation of SFs with CM-SM compared to unstimulated SFs (Figure 4).

The corresponding mRNAs encoding proteins (YKL-40, TSG-6), cytokines (IL-6, IL-8), chemokines (RANTES, MCP-1), MMPs (MMP-1, MMP-3) and ADAMTS-5, associated with OA, were quantified in stimulated and non-stimulated SFs. We found that CM-SM and CM-IFP induced the highest level of pro-inflammatory gene expression in SFs (Figure 5), which is comparable to the results of previous studies. Belluzi et al. also demonstrated, that CM from OA tissues, such as CRT, IFP, meniscus, and synovium, induced and increased IL-8 and CCL21 production in the synoviocyte cell line and confirmed that CM from all OA joint tissues could contribute to the progression of synovitis, with SM playing a predominant role compared to other joint tissues [26]. It was also observed that the co-cultivation of SFs with CM-CRT did not induce increased inflammatory gene expression and the mRNA levels were comparable to those of the control, which is consistent with the results obtained from the protein level analysis.

However, in the case of chemical activators, IFN-γ did not induce increased inflammatory gene expression in SFs. Also, activation by IL-1β and TNF-α resulted in lower inflammatory gene expression levels than in the case of tissue-activated SFs and the change was not statistically significant. A single exception was the increase in gene expression of RANTES, after the stimulation of SFs with IL-1β and TNF-α (Figure 5a). On the contrary, in the study of Haltmayer et al., a co-culture model consisting of osteochondral tissue and SM explants stimulation with IL-1β and TNF-α resulted in an OA-like inflammatory response with increased gene expression of MMPs and IL-6 [41]. Several cell types in the OA synovium could be responsible for the observed synovial inflammation. Studies focused on the depletion of synovial macrophages have shown that IL1-β and TNF-α in the OA synovium are mainly produced by CD14+ synovial macrophages. The depletion of these macrophages leads to a decrease in levels of IL-1β and TNF-α, which in turn may inhibit the release of IL-6, chemokines IL-8, MCP-1 and MMPs (MMP-1 and MMP-3). Thus, macrophages appear to drive the inflammatory and destructive responses of SFs through the combined actions of IL1-β and TNF-α [42].

A significant increase in gene expression of TNF-stimulated gene-6 protein (TSG-6) and Chitinase-3-like protein 1 (CHI3L1), also known as YKL-40, was also observed after stimulation with CM-SM (Figure 5e), and in the case of YKL-40, also after stimulation with CM-IFP (Figure 5f) compared to the control. Many authors have suggested that YKL-40 and TSG-6 could be used as novel biomarkers for joint inflammation and progression of OA in the knee [43,44,45]. TSG-6 is hyaluronan (HA)-binding protein that is stimulated by pro-inflammatory cytokines, growth factors and hormones [46]. In the study by Chou et al., TSG-6 protein and mRNA were strongly expressed in damaged articular and meniscal CRT and cytokine-treated chondrocytes [47]. YKL-40 is a 40 kDa glycoprotein secreted by chondrocytes and synoviocytes. Conrozier et al. found a positive correlation between levels of YKL-40 and CRP in patients with OA [43], and Vaananen, T., et al. confirmed that the levels of intra-articular YKL-40 correlate with the CRT matrix-degrading enzymes MMP-1 and MMP-3 [44]. A significant increase in mRNA levels of MMP-1, MMP-3 and ADAMTS-5 in SFs stimulated with CM-SM, compared to the control, was also observed in our study. MMPs are known to regulate ECM degradation by cleaving the peptide bond of the target proteins. Collagen type II is cleaved by MMPs, whereas aggrecan is cleaved by enzymes of the ADAMTS type, most likely ADAMTS-5. While the loss of aggrecan from CRT is reversible, the degradation of collagen is not and, therefore, represents an irreversible step in CRT degradation [48,49].

## 4. Materials and Methods

### 4.1. Harvesting of OA Tissues and Preparation of CM

Samples of SM, IFP, CRT were collected as surgical waste from OA female patients (*n* = 5) with Kellgren–Lawrence grade III/IV who underwent a total knee replacement. All patients (mean age 72 years; interquartile range (IQR) 12. 5 median of the body mass index (BMI) 30.6 kg/m^2^, IQR 10) fulfilled the criteria for the diagnosis of OA. The study was approved by the ethical committee of the P. J. Safarik University and L. Pasteur University Hospital in Kosice, Slovakia, and was performed after obtaining informed consent from the patients. Joint tissues were transferred into Dulbecco’s modified Eagle’s medium (DMEM, Sigma Aldrich, Steinheim, Germany) supplemented with 1% (*v*/*v*) antibiotic/antimycotic solution containing 100 IU penicillin/mL, 100 μg streptomycin/mL and 0.25 μg amphotericin B/mL (Sigma Aldrich, Steinheim, Germany), carefully washed in phosphate buffer solution (PBS, Sigma Aldrich, Steinheim, Germany), dissected and weighted in order to obtain tissue samples at a ratio ~150 mg/mL media. Tissue samples were incubated in DMEM (Sigma Aldrich, Steinheim, Germany) for 24 h at 37 °C in a humidified atmosphere and 5% of CO_2_. CM of OA tissues (SM, IFP, CRT) was collected, centrifuged at 300× *g* for 10 min at 4 °C to remove cell debris, filtered through a 0.22 µm filter and stored at −80 °C until use.

### 4.2. Isolation and Characterization of SFs

Isolation of SFs was performed as previously described [50]. Briefly, synovial tissue was intensively washed in PBS (Sigma Aldrich, Steinheim, Germany), cut into small pieces and digested with 1.0 mg/mL collagenase type II (Gibco, Bleiswijk, The Netherlands) in low glucose DMEM containing 1% (*v*/*v*) antibiotic/antimycotic solution overnight at 37 °C under continuous rolling. The digested fragments were then filtered through a 70 μm cell strainer (BD Falcon™, Biosciences, NJ, USA). The cell suspension was centrifuged at 300× *g* for 10 min at 4 °C, washed twice in DMEM, resuspended in culture medium containing α-MEM (Sigma Aldrich, Steinheim, Germany), 10% Fetal Bovine Serum (FBS, Gibco, Bleiswijk, The Netherlands), 1% (*v*/*v*) antibiotic/antimycotic solution, 1% L-glutamine (Sigma Aldrich, Germany) and cultured at 37 °C in a humidified atmosphere and 5% of CO_2_. When the cells reached 80% confluence, they were detached with 0.05% Trypsin-EDTA (Gibco, Bleiswijk, The Netherlands) and seeded at a density of 2 × 10^3^ cells/cm^2^. The number and viability of cells were determined using TC10™ Automated Cell Counter (Bio-Rad Laboratories, Hercules, CA, USA). Cells from passage 2 were used for further analysis. The phenotype of SFs was determined by flow cytometry. At least 2 × 10^5^ cells were incubated with either fluorescein isothiocyanate (FITC), phycoerythrin (PE) or allophycocyanin (APC) conjugated antibodies (CD14, CD26, CD34, CD45, CD54, CD73, CD90, CD105, CD106) for 10 min in dark, washed in PBS (Sigma Aldrich, Steinheim, Germany) and centrifuged at 300× *g* for 10 min. The resuspended cell pellets were analyzed on a Becton Dickinson FACSCalibur using CellQuestPro software (Becton Dickinson).

### 4.3. Stimulation of SFs with CM from OA Tissues and Cytokines

SFs were seeded at a density of 2 × 10^4^ cells per well in 6-well culture plates. After about 2 weeks, the cells were carefully washed with PBS (Sigma Aldrich, Steinheim, Germany) and cultured with CM-SM, CM-IFP, CM-CRT separately in a ratio (1:1) with a culture medium for 24 h. The stimulation of SFs was performed similarly with cytokines (IL-1β + TNF-α) and IFN-γ with a final concentration of 10 ng/mL in the culture medium. Unstimulated SFs in culture medium were used as a negative control. After 24 h, the cells were washed twice in PBS (Sigma Aldrich, Steinheim, Germany) and the culture medium was replaced with DMEM without phenol red (Sigma Aldrich, Steinheim, Germany) for another 24 h. The CM obtained after stimulation of SFs with joint OA tissues and cytokines was collected, centrifuged at 300× *g* for 10 min at 4 °C, filtered through a 0.22 µm filter and stored at −80 °C for further analysis. For gene expression analysis, SFs were lysed with TRIzol reagent (ThermoFischer, Waltham, MA, USA). 

### 4.4. The Proliferation Rate of Stimulated SFs

The xCELLigence^®^ RTCA SP device (Roche Applied Science, Penzberg, Germany) was used to continuously monitor the effect of OA joint tissues and cytokines on the proliferation of SFs. The procedure was performed as previously described in our in vitro study [51]. In brief, the cells were seeded at a density of 7 × 10^3^ cells per well in 96-well E-Plate^®^ (Roche Applied Science, Penzberg, Germany). After leaving the plate at room temperature (RT) for 30 min to allow cell attachment, the plate was transferred to the RTCA SP device in the incubator and the impedance value of each well was automatically monitored by the xCELLigence system and expressed as cell index (CI). After approximately 51 h, CM of OA tissue (SM, IFP, CRT) and cytokines (IL-1β + TNF-α, IFN-γ) were added to stimulate SFs. Cell attachment and proliferation were continuously monitored every hour for a period up to 145 h. The proliferation rate was analyzed by RTCA software.

### 4.5. Characterization of CM from OA Tissues and CM from Stimulated SFs by Multiplex Immunoassay

Protein release from OA tissues and stimulated SFs into the CM was evaluated using multiplex bead-based sandwich immunoassay kits. Concentrations of 11 biomolecules (EGF, Eotaxin, FGF-2, IL-10, IL-1Ra, IL-8, IP-10, MCP-1, PDGF-AB/BB, RANTES, VEGF) were quantified at least in duplicate for each sample using MILLIPLEX^®^ Assays (Merck KGaA, Gernsheim, Germany) according to the manufacturer’s protocol and the MAGPIX Luminex platform. Briefly, 25 µL of the standards, controls and samples was added to each well, and then 25 µL of antibody-immobilized beads was added. The plate was incubated overnight at 4 °C, then washed with 200 µL washing buffer and incubated with 25 µL of detection antibodies for 1 h at RT. Finally, the plate was incubated with 25 µL of streptavidin-PE for 30 min at RT, washed and measured in a reader. Next, xPONENT software 4.2 for MAGPIX (Luminex Corporation, Austin, TX) and Bio-Plex Manager 6.1 (Bio-Rad Laboratories, Hercules, CA, USA) were used for data analysis. Once standard curves were generated, concentrations were interpolated for each sample using a 5-parameter curve fitting equation and expressed in pg/mL.

### 4.6. Analysis of Gene Expression by RT-qPCR

Total RNA was isolated from approximately 3 × 10^5^ stimulated and unstimulated (control) SFs cells using TRIzol reagent (ThermoFischer, Waltham, MA, USA) and RNeasy Micro kit (Qiagen, Germantown, MD, USA) following the manufacturer’s protocol. Quality and quantity of RNA samples were analysed using NanoDrop spectrophotometer and agarose gel. Following this, 1 µg of RNA was reverse transcribed using SuperScript™ VILO™ cDNA Synthesis Kit (Invitrogen, Waltham, MA, USA) using oligo(d)T primers according to the manufacturer’s protocol. The obtained cDNA was diluted 10× and used directly in qPCR or stored at −80 °C. Quantitative PCR (qPCR) was performed using PowerUp™ SYBR™ Green Master Mix (Applied Biosystems, San Francisco, CA, USA) on a CFX96 Real-Time Detection System (Bio-Rad, Hercules, CA, USA). A 20 μL reaction contained 1× PowerUp Sybr Green master mix, 200 nM of each forward and reverse primer and 1.5 μL of 10× diluted cDNA. PCR conditions were as follows: 50 °C for 2 min for UDG activation, 95 °C for 2 min for initial denaturation, followed by 40 cycles of 95 °C for 20 s, annealing and extension at 60 °C for 1 min including plate reading. The relative gene expression of the selected target genes was calculated using the 2^−ΔΔCt^ method [52]. Gene expression was normalized to two housekeeping genes (RPL13 and GAPDH), which were previously tested using the geNorm algorithm [53] and selected as the most stable reference genes. Gene expression was calculated as a fold change compared to the untreated control. The list of selected target and housekeeping genes and their primer sequences can be found in Table 2. Primer sequences originate from the website origene.com (accessed on 7 February 2022).

### 4.7. Statistical Analysis

Statistical analyses were performed using one-way ANOVA parametric tests. Protein concentration in CM from OA joint tissues (SM, IFP, CRT) was analyzed by one-way analysis of variance using Bonferroni’s multiple comparison test as post-hoc analysis. Protein concentration, mRNA and proliferation rate of stimulated SFs were analyzed using one-way analysis of variance and Dunn’s multiple comparison test as post-hoc analysis. Statistical analysis was performed using GraphPad Prism 5.0 statistical software (GraphPad Prism Software, Inc., San Diego, CA, USA). Data are presented as the mean ± SD, *p* values less than 0.05 were considered significant (* *p* < 0.05, ** *p* < 0.01, *** *p* < 0.001).

## 5. Conclusions

In our study, it was demonstrated that CM-SM had the most significant effect on SFs and the establishment of an in vitro inflammatory microenvironment. This result was demonstrated by the significantly increased production of pro-inflammatory molecules (IL-6, IL-8, MCP-1, RANTES, VEGF) and by the highest mRNA expression of all tested inflammatory markers compared to SFs controls. Based on our preliminary results, the stimulation of SFs with CM-SM is effective in mimicking the inflammatory and catabolic environment of OA in vitro. Our observations also suggest that not only established signaling molecules, such as recombinant cytokines, but also CM from OA tissues, including inflammatory factors as mediators of communication among different joint cells and tissues, play an important role in OA pathogenesis. The data shown represent a prerequisite for creating an in vitro OA model. In conclusion, our recent work serves as a scientific basis for further analyses of cell interactions, extracellular products examination, and establishment of a 2D OA in vitro model, which better mimics in vivo cell bioactivity and cartilage microenvironment.

## Figures and Tables

**Table 1:** Cell surface marker identification of SFs (passage 2) analyzed by flow cytometry (*n* = 5, mean % ± SD).

Cell Surface Markers	CD14	CD34	CD45	CD106	CD26	CD54	CD73	CD105	CD90
mean % ± SD	1.45 ± 0.46	0.46 ± 0.18	0.28 ± 0.46	4.15 ± 2.38	82.1 ± 20.2	33.6 ± 7.86	99.8 ± 1.48	98.3 ± 0.82	91.9 ± 3.61

**Table 2:** Selected target and housekeeping genes and sequences of primers used in RT- qPCR.

Gene	Forward Primer 5′ → 3′	Reverse Primer 5′ → 3′
*RPL13*	CTCAAGGTGTTTGACGGCATCC	TACTTCCAGCCAACCTCGTGAG
*GAPDH*	GTCTCCTCTGACTTCAACAGCG	ACCACCCTGTTGCTGTAGCCAA
*IL-6*	AGACAGCCACTCACCTCTTCAG	TTCTGCCAGTGCCTCTTTGCTG
*IL-8*	GAGAGTGATTGAGAGTGGACCAC	CACAACCCTCTGCACCCAGTTT
*MMP-1*	ATGAAGCAGCCCAGATGTGGAG	TGGTCCACATCTGCTCTTGGCA
*MMP-3*	CACTCACAGACCTGACTCGGTT	AAGCAGGATCACAGTTGGCTGG
*RANTES*	CCTGCTGCTTTGCCTACATTGC	ACACACTTGGCGGTTCTTTCGG
*MCP-1*	AGAATCACCAGCAGCAAGTGTCC	TCCTGAACCCACTTCTGCTTGG
*TSG-6*	TCACCTACGCAGAAGCTAAGGC	TCCAACTCTGCCCTTAGCCATC
*YKL-40*	CCACAGTCCATAGAATCCTCGG	TGCCTGTCCTTCAGGTACTGCA
*ADAMTS-5*	CCTGGTCCAAATGCACTTCAGC	TCGTAGGTCTGTCCTGGGAGTT

## Data Availability

The study did not report any data.
